# Lack of high BMI-related features in adipocytes and inflammatory cells in the infrapatellar fat pad (IFP)

**DOI:** 10.1186/s13075-017-1395-9

**Published:** 2017-08-11

**Authors:** Anja J. de Jong, Inge R. Klein-Wieringa, Stefan N. Andersen, Joanneke C. Kwekkeboom, Linda Herb-van Toorn, Badelog J. E. de Lange-Brokaar, Danny van Delft, John Garcia, Wu Wei, Huub J. L. van der Heide, Yvonne M. Bastiaansen-Jenniskens, Gerjo J. V. M. van Osch, Annemarie M. Zuurmond, Vedrana Stojanovic-Susulic, Rob G. H. H. Nelissen, René E. M. Toes, Margreet Kloppenburg, Andreea Ioan-Facsinay

**Affiliations:** 1 Department of Rheumatology, Leiden University Medical Centre, C1-R, Albinusdreef 2, 2333 ZA Leiden, The Netherlands; 2 Department of Orthopaedics, Leiden University Medical Center, Leiden, The Netherlands; 3 Department of Orthopaedics, Erasmus MC, University Medical Center, Rotterdam, The Netherlands; 4 ISTM, Keele University, Robert Jones and Agnes Hunt Orthopaedic Hospital, Oswestry, Shropshire UK; 5 TNO, Leiden, The Netherlands; 6 Janssen, Pharmaceutical Companies Johnson & Johnson, Springhouse, Pennsylvania USA

**Keywords:** Osteoarthritis, Infrapatellar fat pad, Obesity, Inflammation, Macrophages

## Abstract

**Background:**

Obesity is associated with the development and progression of osteoarthritis (OA). Although the infrapatellar fat pad (IFP) could be involved in this association, due to its intracapsular localization in the knee joint, there is currently little known about the effect of obesity on the IFP. Therefore, we investigated cellular and molecular body mass index (BMI)-related features in the IFP of OA patients.

**Methods:**

Patients with knee OA (N = 155, 68% women, mean age 65 years, mean (SD) BMI 29.9 kg/m2 (5.7)) were recruited: IFP volume was determined by magnetic resonance imaging in 79 patients with knee OA, while IFPs and subcutaneous adipose tissue (SCAT) were obtained from 106 patients undergoing arthroplasty. Crown-like structures (CLS) were determined using immunohistochemical analysis. Adipocyte size was determined by light microscopy and histological analysis. Stromal vascular fraction (SVF) cells were characterized by flow cytometry.

**Results:**

IFP volume (mean (SD) 23.6 (5.4) mm^3^) was associated with height, but not with BMI or other obesity-related features. Likewise, volume and size of IFP adipocytes (mean 271 pl, mean 1933 μm) was not correlated with BMI. Few CLS were observed in the IFP, with no differences between overweight/obese and lean individuals. Moreover, high BMI was not associated with higher SVF immune cell numbers in the IFP, nor with changes in their phenotype. No BMI-associated molecular differences were observed, besides an increase in TNFα expression with high BMI. Macrophages in the IFP were mostly pro-inflammatory, producing IL-6 and TNFα, but little IL-10. Interestingly, however, CD206 and CD163 were associated with an anti-inflammatory phenotype, were the most abundantly expressed surface markers on macrophages (81% and 41%, respectively) and CD163^+^ macrophages had a more activated and pro-inflammatory phenotype than their CD163^-^ counterparts.

**Conclusions:**

BMI-related features usually observed in SCAT and visceral adipose tissue could not be detected in the IFP of OA patients, a fat depot implicated in OA pathogenesis.

**Electronic supplementary material:**

The online version of this article (doi:10.1186/s13075-017-1395-9) contains supplementary material, which is available to authorized users.

## Background

Obesity is associated with the development and progression of osteoarthritis (OA). This association is not only observed in weight-bearing joints such as the knee, but also in hand OA [1, 2]. These observations suggest that in addition to mechanical factors, systemic factors associated with obesity play a role in the pathophysiology of OA.

Adipose tissue consists of adipocytes and the stromal vascular fraction (SVF), which contains a variety of cells, including immune cells. Obesity is usually accompanied by adipose tissue inflammation, characterized by changes in adipocytes and inflammatory cells, leading to a shift from an anti-inflammatory phenotype to a pro-inflammatory phenotype of the adipose tissue [3]. Adipocytes are generally enlarged in obesity and this growth causes expansion of the adipose tissue, but also adipocyte cell death and hypoxia [4–6]. This process is accompanied by macrophage infiltration and congregation around dead or necrotic adipocytes, resulting in formation of so-called crown like structures (CLS) where macrophages are thought to scavenge adipocyte remnants [7–9]. Furthermore, the polarization state of macrophages in the obese adipose tissue changes towards a more pro-inflammatory phenotype, thereby sustaining and promoting inflammation of the adipose tissue [10–12].

The knee joint is characterized by the presence of an adipose tissue depot called the infrapatellar fat pad (IFP) also known as Hoffa’s fat pad. The IFP is intracapsularly and extrasynovially located in close vicinity to the synovium, cartilage and bone. Due to its localization, it is conceivable that the IFP contributes to the pathophysiology of OA in the joint, through secretion of soluble mediators. Our group and others have shown that the IFP has a pro-inflammatory phenotype in patients with advanced knee OA [13–15] and could thereby contribute to inflammation in the joint (reviewed in [16]).

To date, little is known about the effect of obesity on the IFP. Two magnetic resonance (MR) studies suggested that, unlike subcutaneous tissue, the IFP does not enlarge with obesity [17, 18]. More recent studies addressing this question have shown contrasting results in humans [19, 20], while various body mass index (BMI)-related changes were observed in the IFP in mice [21]. Moreover, we have previously reported differences in the IFP, such as enhanced TNFα secretion, in relation to high BMI [13]. Nonetheless, given the correlation between obesity and OA, it is important to fully comprehend the possible molecular and cellular BMI-related features in fat tissue located in close contact with the joints that are affected in OA, as is the case for the IFP.

Therefore, we extensively investigated the cellular and molecular features of adipose tissue typically associated with obesity to assess whether the IFP changes in OA patients with an increase in BMI and could thereby contribute to disease progression. We determined the association between high BMI and the volume of the IFP, the volume of adipocytes, their cytokine secretion profile, and the number and phenotype of adipose tissue immune cells.

## Methods

### Human subjects

**Fig. 1:**
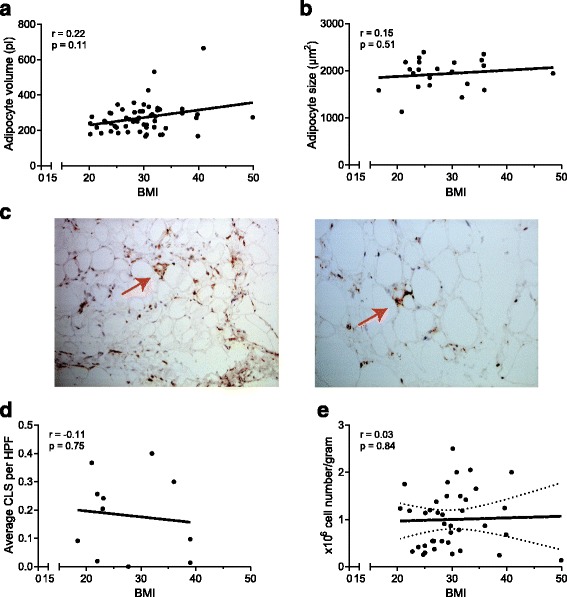
Adipocyte volume, and the number of crown-like structures (*CLS*) and stromal vascular fraction (SVF) cells in the infrapatellar fat pad (IFP) did not correlate with body mass index (*BMI*). Adipocytes were isolated from the IFP from patients with osteoarthritis (OA), who were undergoing total knee-replacement surgery, adipocyte volume was determined and the correlation with BMI was assessed (N = 56) (**a**). Adipocyte size was determined upon haematoxylin and eosin (H&E) staining and the correlation with BMI was assessed (N = 18) (**b**). IFP tissue was stained for CD68 and the number of CLS was quantified. A representative picture of the staining at × 20 (*left*) and × 40 (*right*) magnification (**c**) and the summary of all results is shown (N = 11) (**d**). The number of SVF cells per gram adipose tissue was determined and the correlation with BMI was assessed (N = 39) (**e**). Correlation was tested using Spearman’s rank correlation (**a**, **b**) or Pearson correlation coefficient (**e**). Each *dot* represents one patient. *HPF* high power field

**Fig. 2:**
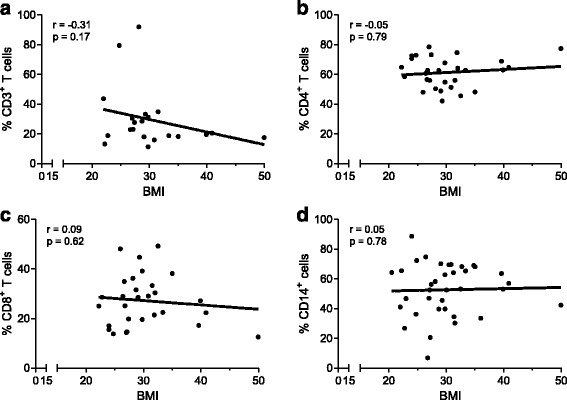
Cell infiltrate in adipose tissue. The stromal vascular fraction (SVF) of the infrapatellar fat pad (IFP) was isolated and T cells and macrophages were characterized by flow cytometry (gating strategies were performed as described in Additional file 1: Figure S3). Percentages of CD3^+^ T cells (N = 21) (**a**), CD4^+^ T cells (N = 29) (**b**), CD8^+^ T cells (N = 29) (**c**) and macrophages (N = 37) (**d**) and their correlation with body mass index (*BMI*) was determined using Spearman’s rank correlation. A *P* value <0.05 was considered significant. Each *dot* represents one patient

**Fig. 3:**
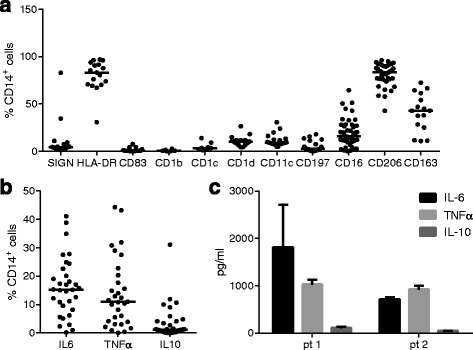
Phenotypic characterization of macrophages in the infrapatellar fat pad (IFP). The stromal vascular fraction (SVF) was isolated from the IFP and macrophages were characterized by flow cytometry. Percentages of CD14^+^ macrophages positive for each specified marker are depicted (N = 6–45) (**a**). *Ex vivo* intracellular cytokine production by CD14^+^ macrophages in the IFP is depicted (N = 13) (**b**). Cytokines were measured in supernatant of unstimulated CD14^+^ from the SVF (N = 2) (**c**). Median (**a** and **b**) or mean (**c**) is indicated; each *dot* represents one patient. *pt* patient

**Fig. 4:**
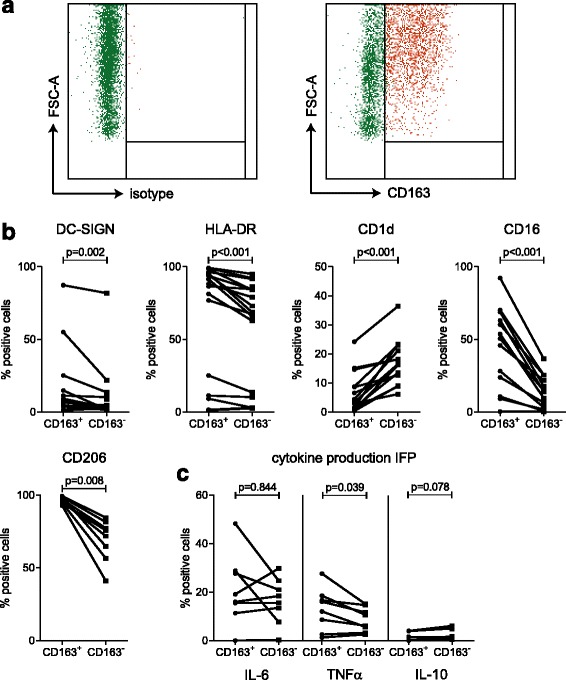
CD163^+^ macrophages in the infrapatellar fat pad (*IFP*) are pro-inflammatory. Total macrophage population (*left*), and CD163^+^ (*red*) and CD163^-^ CD14^+^ (*green*) macrophages (*right*) are depicted against the forward scatter-area (*FSC-a*) (**a**). Differences between CD163^+^ and CD163^-^ CD14^+^ macrophages in surface marker expression (N = 7–12) (**b**) and *ex vivo* intracellular cytokine production (N = 7–8) (**c**). Each *line* indicates one patient sample. A *P* value <0.05, determined by the Wilcoxon signed rank test (**b**) or paired Student’s *t* test (**c**) was considered significant

In total, 155 patients with knee OA were recruited in the study. The patients were 68% women, with mean age 65 years and mean (SD) BMI 29.9 (5.7) kg/m^2^. The distribution of the patients among BMI categories were: underweight (BMI below 18.5), none; normal (BMI 18.5–24.9), 28 patients; overweight (25.0–29.9), 61 patients; and obese (30.0 and above), 66 patients. Characteristics of the patients represented in Figs. 1, 2, 3 and 4 are shown in Additional file 1: Table S1. A total of 102 patients were part of the GEneration of Models, Mechanisms & Markers for STratification of OsteoArthritis patieNts (geMstoan) study, an observational study in patients with knee OA to find new biomarkers for OA progression. The patients were included between 2008 and 2014, had symptomatic knee OA, following the American College of Rheumatology (ACR) criteria [22], and attended the orthopaedic department of the Leiden University Medical Centre (LUMC) or the orthopaedic department of the Alrijne Ziekenhuis in Leiden. Written informed consent is available from all geMstoan patients. Of these 102 patients who participated in the geMstoan study, a total of 79 patients underwent magnetic resonance imaging (MRI), 30 of whom underwent joint-replacement surgery and 49 of whom underwent arthroscopy. IFPs and subcutaneous adipose tissue (SCAT) were obtained from 53 patients participating in geMstoan, who were undergoing joint replacement surgery after giving informed consent. Leftover IFP and SCAT were obtained during arthroplasty from an additional 23 patients from the LUMC, and 30 patients from Erasmus Medical Centre (MC). SCAT was obtained from the thigh, next to the incision made for the total knee-replacement surgery. Diagnosis, age, gender and BMI were available for the latter patients. The study was approved by the local medical ethical committee. Consent was given in accordance with the guidelines of the Federation of Biomedical Scientific Societies (http://www.federa.org) after approval by the local ethical committee (MEC 2008-181 and MEC 2012-267). Not all experiments could be performed with each tissue sample as the sample size was limited.

### MRI acquisition

We used a 3 T Philips Achieva MR system (Philips Healthcare, Best, The Netherlands) with an 8-channel dedicated knee coil. Sagittal proton density (PD) fast spin-echo (FSE)-driven equilibrium images were obtained with a field of view (FOV) of 150 × 150 mm, an acquisition matrix of 304 × 240, slice thickness of 3 mm, repetition time (TR) of 3000 ms and echo time (TE) of 34 ms. Subsequently, contrast enhanced (CE) MR images were obtained following injection of gadoterate meglumine (0.2 ml/kg) (Dotarem; Guerbet) into the cubital vein using a power injector (Medrad) with a rate of 2 ml/s followed by a 40-ml saline flush also at a rate of 2 ml/s. We subsequently obtained frequency selective fat-suppressed T1-weighted, FSE with TR of 655 ms, and TE of 20 ms, in both the axial and sagittal planes.

### IFP volume determination by MRI

IFP volume was measured by manual segmentation of IFP boundaries on section-by-section sagittal PD FSE images, using the software program OsiriX. T1-weighted CE images were used to distinguish and compare between IFP and non-IFP structures (Additional file 1: Figure S1). The software program OsiriX measured IFP volume by making a 3D model of the drawn contours. Two independent observers measured IFP volume on all MRI scans. The intraclass correlation (ICC) was 0.957 for intra-observer reliability (measured in all images). The ICC was 0.909 for inter-observer reliability (measured in all images).

### Measurement of adipocyte size

Adipocytes were isolated as previously described [13]. Briefly, adipose tissue was digested with collagenase type 1A (Sigma-Aldrich) for 1 h and the tissue was filtered through a 250-μm nylon mesh. Adipocytes were washed three times with medium (DMEM/F12 supplemented with 0.5% free fatty acid (FFA) free bovine serum albumin (BSA), 15 mM Hepes, 2 mM glutamax and 100 U/ml penicillin/streptomycin) by allowing them to float to the surface, followed by removal of medium and addition of fresh medium. The diameter of 100 adipocytes was determined with light microscopy with an ocular micrometer and the mean volume was calculated, based on the formula:

V = πd^3^/6,

where d is the diameter of the adipocyte, and the mean volume of adipocytes was then calculated. Explants of the IFP and SCAT were also cryosectioned and stained with haematoxylin and eosin (H&E) and imaged using an Olympus SC30 camera (Olympus, Zoeterwoude, The Netherlands). The cross-sectional area of the imaged adipocytes was calculated using Fiji Is Just ImageJ software with the additional Adiposoft plugin. Three separate sections, with a minimum of 25 adipocytes in each section were measured per donor. The Adiposoft application was calibrated to identify cells with a diameter between 30 and 130 μm. A 0.33 μm/pixel measuring-scale was also used by the application to determine the cross-sectional area (size) of each adipocyte identified in the images. A manual inspection of output data was performed to confirm the consistency of the measurements.

### Histological staining

Pieces of IFP were fixed in 4% formalin overnight followed by storage in EtOH, before embedding in paraffin. Sections of 4 μm at different depths in the tissue were deparaffinised in xylene (Merck, Germany) and EtOH. Endogenous antigens were peroxidised in a MetOH/H_2_O_2_ solution, and antigen retrieval was performed with EDTA (DAKO, USA) at 96 °C for 30 min. After cooling, two consecutive coupes of three different layers were stained for mouse anti-human CD68 (clone KP-1 1:800, DAKO, USA) or the isotype control (mIgG1 (DAKO, USA)) for 1 h at room temperature (RT). CD68^+^ cells were then visualized using DAKO EnVision and DAB Ni kits, according to manufacturers’ instructions (DAKO, USA; Vector Laboratories, Canada) before counterstaining with haematoxylin (Klinipath, The Netherlands). Slides were then embedded in pertex (Histolab Products, Sweden) and analysed using a Leica microscope and Leica software. Two to three slides per patient were used and a total of 11–37 high power field (HPF) pictures were taken depending on the size of the tissue. Two independent scorers (AJJ and SNA) quantified the amount of CLS (the ICC for inter-observer reliability was 0.985) in each HPF and an average of both scorers was generated for the amount of CLS per HPF.

### SVF isolation

The stromal vascular fraction (SVF) was isolated from the IFP and SCAT as previously described [13]. SVF cells were counted by light microscopy, surface staining was performed and the remaining cells were plated overnight in a 6-well plate at a density of maximal 5 × 10^6^ cells/well in medium (DMEM/F12 supplemented with 0.5% FFA free BSA, 15 mM Hepes, 2 mM glutamax and 100 U/ml penicillin/streptomycin) supplemented with 50 IU/ml IL-2 (Peprotech, USA) and 3 μg/ml Brefeldin A (Sigma Aldrich, Germany). Thereafter, cells were harvested using a cell scraper and surface and intracellular staining was performed. The SVF of the IFP from two patients was used to isolate CD14^+^ cells using magnetic-labelled anti-CD14 beads (Miltenyi Biotec) according to the manufacturer’s instructions. CD14^+^ cells were plated in a 96-well plate and supernatant was harvested after 2 days of culture.

### Flow cytometric analysis

Approximately 100,000 freshly isolated SVF cells were stained for 30 min at 4 °C with surface antibody (Ab) solutions containing mixes of the following Abs: PE-conjugated CD1d, CD3, DC-SIGN, CD206 and CD16; FITC-conjugated HLA-DR, CD11c and CD45; APC-conjugated CD8 and CD163; PE-Cy-7-conjugated CD14, PB-conjugated CD4 and Pe-Cy5-conjugated CD206 (all Abs from BD biosciences, except Ab to CD163 and CD206, which were from Biolegend). When specified, approximately 400,000 SVF cells harvested after overnight incubation with brefeldin A were stained for 30 min at 4 °C for surface markers and intracellular cytokines using the BD intracellular cytokine fixation/permeabilization solution kit (BD Biosciences) according to the manufacturer’s instructions. The following Abs were used: PE-Cy7-conjugated CD14; APC-conjugated CD163; PE-conjugated IL-10, TNFα and IL-6 (all Abs from BD biosciences, except Ab to IL-6 which was from eBioscience). Exclusion of dead cells was performed in all experiments using the Dead Cell discrimination kit (Miltenyi Biotec, Germany). Cells were fixed with 1% paraformaldehyde and analysed with a LSR II flow cytometer using Diva 6 software (BD biosciences).

### Generation of fat-conditioned and adipocyte-conditioned medium

Fat-conditioned and adipocyte-conditioned medium (FCM and ACM, respectively) were obtained as previously described [13]. Briefly, FCM was obtained by culturing 100 mg/ml of small pieces of IFP or SCAT in 6-well plates in medium (DMEM/F12 supplemented with 0.5% FFA free BSA, 15 mM Hepes, 2 mM glutamax and 100 U/ml penicillin/streptomycin). Medium was refreshed after 2 h and supernatant was collected after 24 h. For ACM, 100 μl/ml of adipocytes were cultured in 6-well plates in medium for 24 h. Supernatants were collected and stored at −80 °C until use.

### Milliplex MAP analysis

Cytokines were measured in supernatants of CD14^+^ cells isolated from SVF and in FCM and ACM using the Milliplex Human Cytokine/Chemokine kit (Millipore), the Bio-Plex array reader and Bio-Plex software in accordance with the manufacturer’s instructions.

### Statistical analysis

Associations between IFP volume measured by MRI and patient characteristics were determined by linear regression analysis after establishing that the assumptions underlying linear regression analysis were met. Both univariate and multivariate linear regression analyses were performed to determine which patient characteristics were associated with IFP volume. The unpaired or paired Student’s *t* test (for normally distributed variables) or the Mann-Whitney test or Wilcoxon’s matched-pairs signed rank test (for non-Gaussian distributions) was used to compare differences between groups. Correlation was tested by calculating the Pearson correlation coefficient (for normally distributed variables) or by using Spearman’s ranked correlation test (for non-Gaussian distributions), as specified. For Pearson correlation, the trend line with 95% confidence interval was depicted. Correction for multiple testing was performed using Bonferroni’s correction. A *P* value ≤0.05 was considered statistically significant.

## Results

### IFP volume is associated with gender and height but not with BMI

To obtain insight into whether the volume of the IFP is associated with obesity-related features or other patient characteristics, we made use of a clinically well-characterized population of patients participating in the geMstoan study (N = 79). Patient characteristics are shown in Table 1. The mean (SD) IFP volume was 23.6 (5.4) mm^3^. Univariate linear regression analysis indicated that BMI is not associated with IFP volume (Table 2). Other obesity-related features such as waist-to-hip ratio (*R* ^2^ = 0.09) and fat percentage (*R* ^2^ = 0.19) were associated with IFP volume, while waist circumference was not. Moreover, IFP volume was associated with male gender (*R* ^2^ = 0.37), height (*R* ^2^ = 0.47) and weight (*R* ^2^ = 0.07), but not with age, nor with radiographic damage (Kellgren-Lawrence (KL) scores) (Table 2). Multivariate linear regression analysis in which all factors that were significantly associated with IFP volume were included indicated that only height remained independently associated with IFP volume (Table 2). The association with gender was still present, but was no longer significant. To understand how the factors included in the multivariate analysis vary with height and gender, we included height or gender as covariates in the linear regression analysis. These analyses indicate that gender is a confounder for most of the observed associations and that only height remained significantly associated with IFP volume upon correction for gender (Table 2). Stratification based on gender indicated that height is associated with IFP volume in women but not in men (data not shown). Of all patients, 17.9% had cardiovascular comorbidities, indicating the presence of metabolic complications. There was no association between IFP volume and BMI upon stratification for the presence of these comorbidities (data not shown).

**Table 1:** Characteristics of patients (N = 79) in whom infrapatellar fat pad volume was measured

Parameters	Value
Age, year, mean (SD)	62.1 (7.5)
Female, *N* (%)	54 (68.4)
BMI, kg/m^2^, mean (SD)	29.4 (5.2)
Height, cm, mean (SD)	170 (9.0)
Weight, kg (SD)	85 (16.4)
Kellgren and Lawrence grade, *N* (%)^a^	
Grade 1	11 (14.1)
Grade 2	21 (26.9)
Grade 3	25 (32.1)
Grade 4	21 (26.9)

*BMI* body mass index

^a^N = 78 patients included in the analysis of Kellgren and Lawrence grade

**Table 2:** Association between patient characteristics and IFP size measured on MRI

Variable	Univariate		Multivariate		Covariate height		Covariate gender	
	*B* (95% CI)	*P* value	*B* (95% CI)	*P* value	*B* (95% CI)	*P* value	*B* (95% CI)	*P* value
Age	-0.07 (-0.2; 0.1)	0.417			0.06 (-0.1; 0.2)	0.369	-0.08 (-0.2; 0.06)	0.258
Gender	6.93 (4.8; 9.0)	<0.001	4.24 (-0.8; 9.3)	0.098	3.33 (0.9; 5.7)	0.006		
BMI	-0.14 (-0.4; 0.1)	0.258			-0.05 (-0.2; 0.1)	0.560	-0.06 (-0.2; 0.1)	0.549
Waist-to-hip ratio	19.8 (5.9; 33.7)	0.006	-6.32 (-20.4; 7.7)	0.373	4.72 (-6.7; 16.1)	0.412	-9.5 (-24.7; 5.9)	0.222
Waist circumference	0.04 (-0.06; 0.1)	0.391			0.003 (-0.1; 0.1)	0.932	-0.02 (-0.1; 0.06)	0.682
Fat percentage	-0.29 (-0.4; -0.2)	<0.001	0.01 (-0.3; 0.3)	0.943	-0.13 (-0.3; -0.1)	0.024	0.02 (-0.2; 0.2)	0.824
Height	0.41 (0.3; 0.5)	<0.001	0.31 (0.2; 0.4)	<0.001			0.29 (0.2; 0.4)	<0.001
Weight	0.08 (0.01; 0.2)	0.023	-0.02 (-0.1; 0.1)	0.773	-0.02 (-0.1; 0.1)	0.475	0.04 (-0.02; 0.1)	0.207
KL score	0.28 (-0.9; 1.5)	0.648			-0.29 (-1.2; 0.6)	0.523	-0.5 (-1.5; 0.5)	0.291

*IFP* infrapatellar fat pad, *MRI* magnetic resonance imaging, *BMI* body mass index, *KL* Kellgren and Lawrence

### Adipocyte volume, and the number of CLS and SVF cells in the IFP did not correlate with BMI

Next, we investigated adipocytes and SVF cells in the IFP. The average volume of adipocytes was 271 pl (Fig. 1a), and the average size was 1933 μm (Fig. 1b). Neither the volume nor the size of adipocytes correlated with BMI. Furthermore, the distribution of adipocyte sizes was not different between lean and obese individuals. Macrophage staining indicated that CLS (Fig. 1c) were present in small numbers in the IFP (mean = 0.18 CLS/HPF) and did not correlate with BMI (Fig. 1d). Likewise, the number of SVF cells in the IFP did not correlate with BMI (Fig. 1e). In contrast, we did observe BMI-related differences in adipocyte volume (Additional file 1: Figure S2a) and size (Additional file 1: Figure S2b-c), and the number of SVF cells (Additional file 1: Figure S2d) in SCAT, a control adipose tissue. Direct comparison between the IFP and SCAT revealed that IFP adipocytes are smaller compared to SCAT adipocytes (Additional file 1: Figure S2e-f), but IFP has more SVF cells compared to SCAT (Additional file 1: Figure S2g).

### Cell infiltrate in adipose tissue

Next, we investigated the type of immune cells in the IFP. Therefore, we determined the percentage of macrophages and T cells in the SVF from the IFP, since these cell types are the most abundant immune cells in adipose tissue. Our data indicated that there was no correlation between CD3^+^ T cells (Fig. 2a), CD4^+^ T cells (Fig. 2b), CD8^+^ T cells (Fig. 2c) or macrophages (Fig. 2d) and BMI, suggesting no BMI-related differences in the types of immune cells in the IFP.

Previously, we found that TNFα secretion by FCM but not ACM correlated with BMI [13]. Using a different cohort of patients, we now showed that FCM derived from the IFP of patients with a high BMI (BMI >30) had higher levels of TNFα compared to FCM derived from the IFP of patients with a low BMI (BMI ≤25), confirming our previous data (Additional file 1: Table S2). In line with our previous findings, levels of TNFα in ACM did not differ between these high and low BMI groups (Additional file 1: Table S2). We further expanded our analyses to a broad range of cytokines. These studies indicated that several cytokines, such as interferon (IFN)α, IL-2, IL-3, IL-4, IL-5, IL-9, IL-13, IL-15, IL-17, platelet-derived growth factor (PDGF)-ABBB, sCD40L, transforming growth factor (TGF)α and TNFβ were undetectable in most FCM and ACM samples. Moreover, FCM had higher levels of granulocyte macrophage-colony stimulating factor (GM-CSF), IL-8 and monocyte chemotactic protein 3 (MCP3) in the group with BMI >30 compared to the group with BMI ≤25 (Additional file 1: Table S3). Likewise, although ACM contained very low levels of TGFα, these seemed to be lower in the group with BMI >30 compared to the group with BMI ≤25 (Additional file 1: Table S4). These differences, however, were no longer significant after correction for multiple testing.

### Phenotypic characterization of macrophages in the IFP

Because macrophages are abundant in the adipose tissue and their phenotype has been shown to change with obesity, we investigated the phenotype of macrophages in the IFP. Virtually all macrophages expressed human leukocyte antigen (HLA)-DR, while CD1c, CD1d, CD11c and DC-SIGN were present on a low percentage of cells (Fig. 3a). Furthermore, among the surface molecules associated with a pro-inflammatory macrophage phenotype, CD16 was the most abundantly expressed, being present on approx. 20% of macrophages, while 80% of the macrophages expressed the mannose receptor CD206, a surface molecule associated with an anti-inflammatory phenotype. Furthermore, less than half of the macrophages expressed the scavenger receptor CD163. None of the surface markers correlated with BMI.

Intracellular cytokine staining revealed that macrophages from the IFP produced mainly IL-6 and TNFα, but little IL-10 directly *ex vivo* (Fig. 3b), indicating a predominantly pro-inflammatory phenotype. No correlation with BMI was observed for any of these cytokines (data not shown). The intracellular cytokine findings were confirmed in culture supernatants of isolated macrophages from two patients (Fig. 3d). Furthermore, these analyses revealed that CD14^+^ cells from the IFP were also capable of secreting eotaxin, fibroblast growth factor-2 (FGF-2), Flt3-ligand, fractalkine, growth-regulated oncogene (GRO), granulocyte colony stimulating factor (G-CSF), GM-CSF, IFNα2, IFNγ, IL-1α, IL-1β, IL-1RA, IL-7, IL-8, IL-12p40, IP-10, MCP-1, MCP-3, MDC, macrophage inflammatory protein (MIP)1α, MIP1β, RANTES, sCD40L, sIL-2Rα, TNFβ and vascular endothelial growth factor (VEGF) (data not shown).

### CD163^+^ macrophages in the IFP are pro-inflammatory

Surface marker expression indicated an anti-inflammatory phenotype of IFP macrophages, while the cytokine production profile suggests that pro-inflammatory macrophages are predominant. However, a restricted percentage of macrophages secrete IL-10, indicating them as anti-inflammatory. The scavenger receptor CD163 has been associated with the resolution of inflammation and tissue regeneration [23–25], but also with inflammation [26–31]. This indicates that the phenotype of CD163^+^ macrophages is unclear. Therefore, we set out to investigate whether the CD163^+^ macrophages comprise the anti-inflammatory macrophage population and compared them to their CD163^-^ counterparts. Flow cytometric characterization indicated that CD163^+^ macrophages are bigger than their CD163^-^ counterparts (Fig. 4a). Furthermore, we found that the percentages of cells positive for CD16, CD206, DC-SIGN and HLA-DR were higher, while the percentage of cells positive for CD1d was lower in CD163^+^ macrophages compared to their CD163^-^ counterparts (Fig. 4b). Additionally, CD163^+^ macrophages were more often positive for IL-6 and TNFα than for IL-10 and seemed to be more positive for TNFα than CD163^-^ macrophages (Fig. 4c). These data indicate that although CD163 is regarded as an anti-inflammatory macrophage marker, CD163^+^ macrophages from the IFP display a pro-inflammatory phenotype.

## Discussion

In this study, we investigated cellular and molecular BMI-related features in the IFPs from OA patients. Our data indicate no substantial differences in the volume of the IFP, adipocyte size or in immune cell numbers and types in relation according to BMI. These data are important as they indicate differential regulation of BMI-related features in different fat tissues. Molecularly, we confirmed the previously reported increase in TNFα secretion in the high-BMI group, but did not find additional significant differences in other cytokines tested. Extensive characterization of macrophages present in the IFP indicated that most cells bear surface markers associated with an anti-inflammatory phenotype (CD206 and CD163), while they secrete predominantly pro-inflammatory cytokines (TNFα and IL-6). Comparisons between CD163^+^ and CD163^-^ macrophages in the IFP indicated that CD163^+^ are pro-inflammatory, larger and more activated than CD163^-^ macrophages.

A high BMI does not necessarily reflect obesity; however, in our geMstoan population, waist circumference and fat percentage both correlated well with BMI (data not shown), indicating that high BMI does reflect obesity in this population of patients. Furthermore, the presence of cardiovascular comorbidities in the obese is higher as compared to lean individuals indicating a high BMI is accompanied by metabolic complications in our population. Although we cannot formally assess this for the other patients included in this study, they are comparable to the geMstoan patients (age above 50 years, all diagnosed with OA), suggesting that a high BMI in these patients also reflects obesity rather than high muscle content.

Our finding that IFP volume determined by MRI is not associated with BMI is in line with previous published data [17, 18]. Other obesity-related factors such as waist-to-hip ratio, fat percentage, weight and the presence of cardiovascular comorbidities were not associated with IFP volume, were no longer significant in the multivariate analysis or when corrected only for gender (data not shown), in line with known gender differences in these obesity-related features [32]. Together, these data support our conclusion that IFP volume is not associated with obesity.

In contrast, we found that height and gender are associated with IFP volume, in agreement with previous studies [18, 33–35]. Multivariate analysis indicated, however, that only height is independently associated with IFP volume, while the association with gender is partially explained by height, leading to a strongly diminished effect size when compared to univariate analysis. Moreover, the association was no longer significant, possibly reflecting a lack of sufficient power. Furthermore, stratification based on gender indicated that height is only associated with IFP volume in women. Although the mechanisms underlying the association between gender and IFP volume remain unknown, it is conceivable that sex hormones could play a role in this as they have been previously described to direct fat storage to different anatomical locations [36]. In contrast to a previous study [17], we did not find an association between IFP volume and age, possibly due to differences in the investigated OA populations, and in the analyses performed.

Previously published data indicate that obesity is accompanied by enlargement of adipocytes [5, 6] and inflammatory cell infiltration in conventional adipose tissues [12, 37, 38]. However, these BMI-related features do not seem to be present in the IFP, although we observed these features in SCAT. Moreover, we did not find an association between any type of inflammatory cells and BMI. However, due to the small sample size available for CD3^+^ T cells the power of this analysis is limited and the lack of association should be interpreted with care. Although a recent study indicated that obesity-related changes such as in adipocyte size and cellular infiltrate occur in the IFP [19], our observations are in line with other studies indicating that the IFP is not enlarged in obesity [17] and does not have obesity-related features [20], supporting earlier findings that the IFP is different from SCAT [13, 20].

The lack of enlargement in the IFP with obesity could be due to its localization inside the knee capsule, which could strongly limit its growth. The question remains, however, as to how the IFP deals with the metabolic stress (i.e. nutrient overload) that accompanies obesity. One could speculate that IFP adipocytes are less metabolically active and are rather inefficient in removing free fatty acids from the circulation and storing them in lipid droplets. Therefore, IFP adipocytes, unlike adipocytes in other adipose tissues, are not enlarged with obesity. Moreover, this could explain the very small number of CLS and the lack of BMI-related increase in inflammatory cells in the IFP, as IFP adipocytes might undergo less apoptosis/cell death, which are the primary events in adipose tissue inflammation. However, this remains to be investigated. Moreover, our data are relevant as they indicate that understanding of the biology of fat tissue located in close contact with joints affected by OA is important to better comprehend the association between obesity and OA, but caution should be taken in translating findings observed in fat tissues to the IFP, a fat tissue implicated in OA pathogenesis.

Our data indicated that macrophages from the IFP have a dual phenotype, with surface markers associated with an anti-inflammatory phenotype and more pro-inflammatory cytokine profiles. Almost all IFP macrophages expressed CD206, a marker generally associated with M2-like macrophages and tissue-resident macrophages. The phenotype and function of CD163^+^ macrophages is unclear, as CD163^+^ macrophages have been associated with wound healing and the resolution of inflammation [23–25], but have also been implicated in inflammation, such as in spondyloarthritis [26–29], psoriasis [29, 30] and inflammatory bowel disease [27, 31]. Our data are in line with previous publications showing that macrophages from SC and omental adipose tissue express both CD163 and CD206 and display a pro-inflammatory cytokine profile [39].

Studies in mice suggest that obese adipose tissue becomes infiltrated with macrophages, which form CLS, take up lipids and acquire a more pro-inflammatory phenotype [40, 41]. In the IFP, CD163^+^ macrophages appeared to have a more activated state compared to CD163^-^ macrophages and were larger than CD163^-^ macrophages. This could indicate that CD163^+^ macrophages are scavenging dead adipocytes, thereby acquiring more lipids. Supporting our hypothesis, a recent study in mice showed that phagocytosis by macrophages leads to upregulation of both CD206 and CD163 [42].

We did not observe obesity-related differences in TNFα production by macrophages (data not shown). This is surprising in view of the finding that the IFP in obese individuals secretes more TNFα [13] and that macrophages are the most abundant cell type in the SVF. Differences in the regulation of secretion vs production could explain the discrepancy. Alternatively, it is possible that other cells, albeit less numerous, would secrete more TNFα than macrophages [43] and this secretion could be modulated by obesity. Finally, it is possible that dissociation of cells from the tissue could influence their cytokine production.

## Conclusions

In conclusion, no cellular BMI-related features previously reported in other adipose tissues were found in the IFPs of OA patients with regard to the IFP volume, adipocyte volume and size, CLS, immune cell numbers or type. These data confirm our previous findings that the IFP is different to SCAT and raise the intriguing possibility that the IFP might be a different type of fat than the conventional SCAT and visceral adipose tissues.

## Additional file

Additional file 1: Figure S1. IFP volume determination. **Figure S2.** Adipocyte volume and size and number of SVF cells in SCAT correlate with BMI. **Figure S3.** Gating strategy. **Table S1.** Patient characteristics for Figs. 1, 2, 3 and 4 and Figure S2. **Table S2.** Levels of TNFα in FCM and ACM. **Table S3.** Levels of cytokines/chemokines in FCM. **Table S4.** Levels of cytokines/chemokines in ACM. (DOCX 4424 kb)

## Abbreviations

Ab: Antibody
ACM: Adipocyte-conditioned medium
ACR: American College of Rheumatology
BMI: Body mass index
BSA: Bovine serum albumin
CE: Contrast enhanced
CLS: Crown-like structures
DMEM: Dulbecco’s modified Eagle’s medium
FCM: Fat-conditioned medium
FFA: Free fatty acid
FOV: Field of view
FSE: Fast spin-echo
G-CSF: granulocyte colony stimulating factor
GeMstoan: GEneration of Models, Mechanisms & Markers for STratification of OsteoArthritis patieNts
GM-CSF: Granulocyte macrophage-colony stimulating factor
GRO: growth-regulated oncogene
H&E: Haematoxylin and eosin
HLA: Human leukocyte antigen
HPF: High power field
ICC: Intraclass correlation
IFN: Interferon
IFP: Infrapatellar fat pad
IL: Interleukin
KL: Kellgren-Lawrence
LUMC: Leiden University Medical Centre
MCP: Monocyte chemotactic protein
MR: Magnetic resonance
MRI: Magnetic resonance imaging
OA: Osteoarthritis
PD: Proton density
SCAT: Subcutaneous adipose tissue
SVF: Stromal vascular fraction
TE: Echo time
TGF: Transforming growth factor
TNF: Tumour necrosis factor
TR: Repetition time
