# Supplementary material for: Lack of high BMI-related features in adipocytes and inflammatory cells in the infrapatellar fat pad (IFP)
Source: Arthritis Res Ther. 2017 Aug 11;19:186. doi: 10.1186/s13075-017-1395-9 (PMC5553811; doi:10.1186/s13075-017-1395-9)
Supplement: Additional file 1: Figure S1. — IFP volume determination. Figure S2. Adipocyte volume and size and number of SVF cells in SCAT correlate with BMI. Figure S3. Gating strategy. Table S1. Patient characteristics for Figs. 1, 2, 3 and 4 and Figure S2. Table S2. Levels of TNFα in FCM and ACM. Table S3. Levels of cytokines/chemokines in FCM. Table S4. Levels of cytokines/chemokines in ACM. (DOCX 4424 kb) [file 13075_2017_1395_MOESM1_ESM.docx]

**Additional file 1**


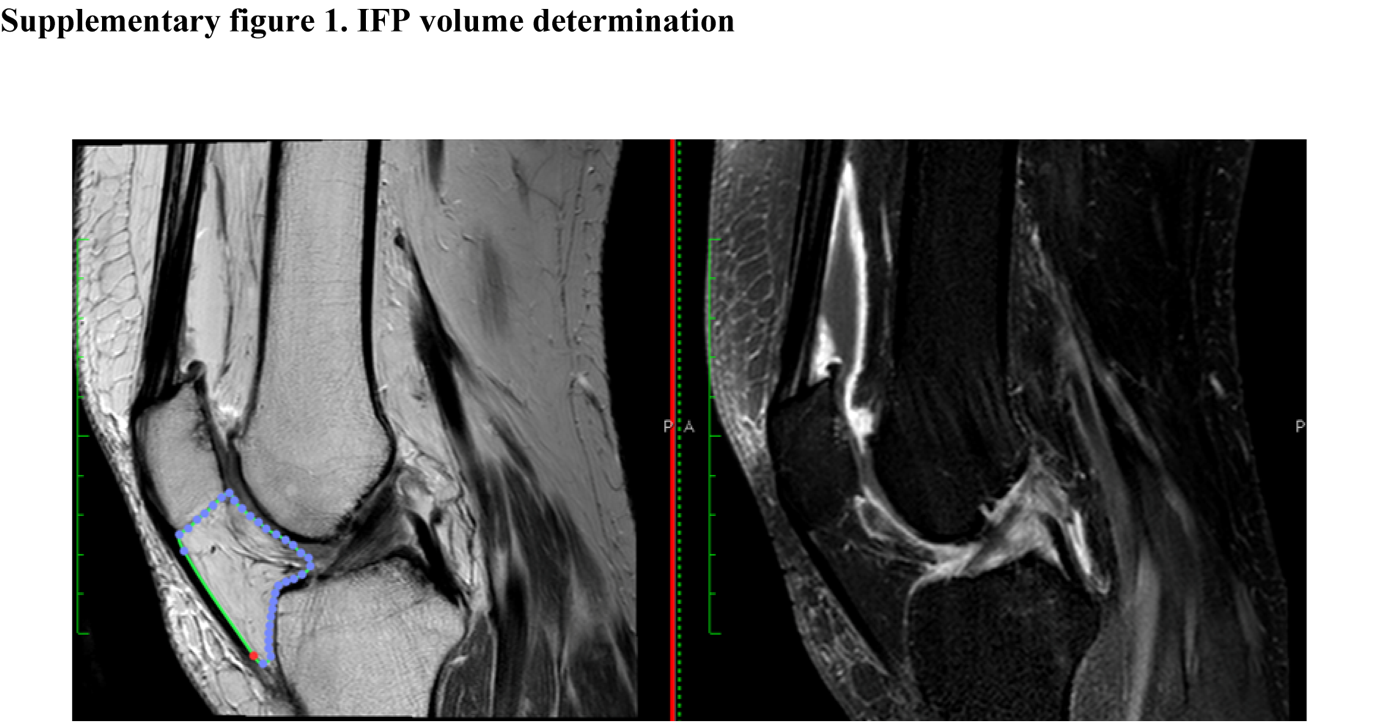


**Figure S1. IFP volume determination**

IFP volume was measured on section-by-section sagittal PD FSE images (left), T1-weighted CE-images were used to distinguish and compare between IFP and non-IFP structures (right).


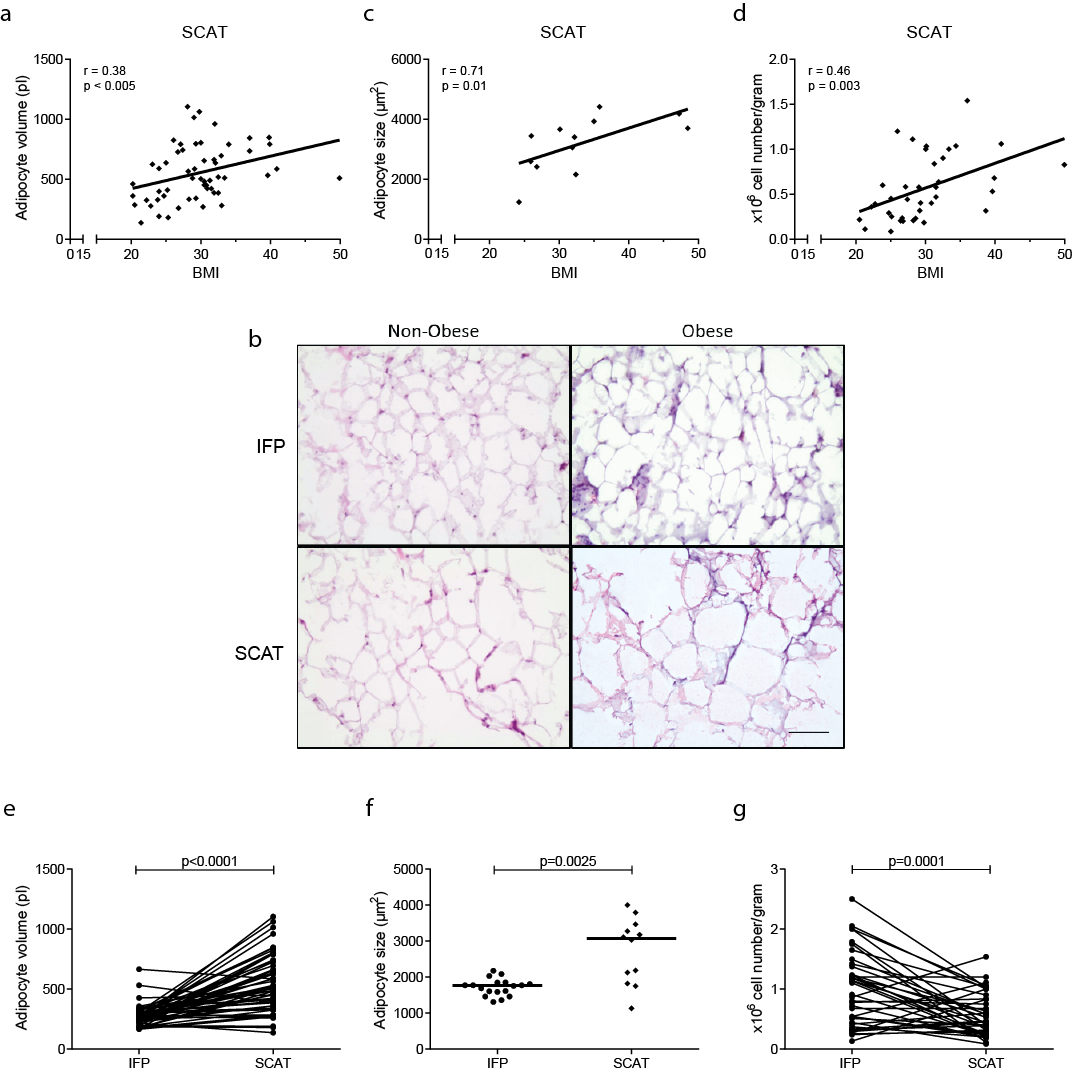


**Figure S2. Adipocyte volume and size and number of SVF cells in SCAT correlate with BMI**

Adipocytes were isolated from control SCAT of OA patients and adipocyte volume was determined and correlated to BMI, N=56 (a). SCAT tissue was H&E stained and adipocyte size was determined. Representative images of H&E stained cryosections of adipose tissue from both the IFP and SCAT tissue of non-obese and obese donors (b). Scale bar= 100μm. The size of adipocytes from SCAT were correlated with BMI, N=12 (c). Number of SVF cells per gram SCAT was determined and correlated with BMI, N=38 (d). A comparison between IFP and SCAT tissue for adipocyte volume (e), size (f) and number of SVF cells per gram (g) is given. Correlations were determined using Spearman’s rank correlation (a, c) or Pearson correlation coefficient (d). P-values for differences between groups (as indicated) were tested by Wilcoxon signed rank test (e), unpaired student’s t-test (f) or paired student’s t-test (g). Medians are depicted and each dot represents one patient.


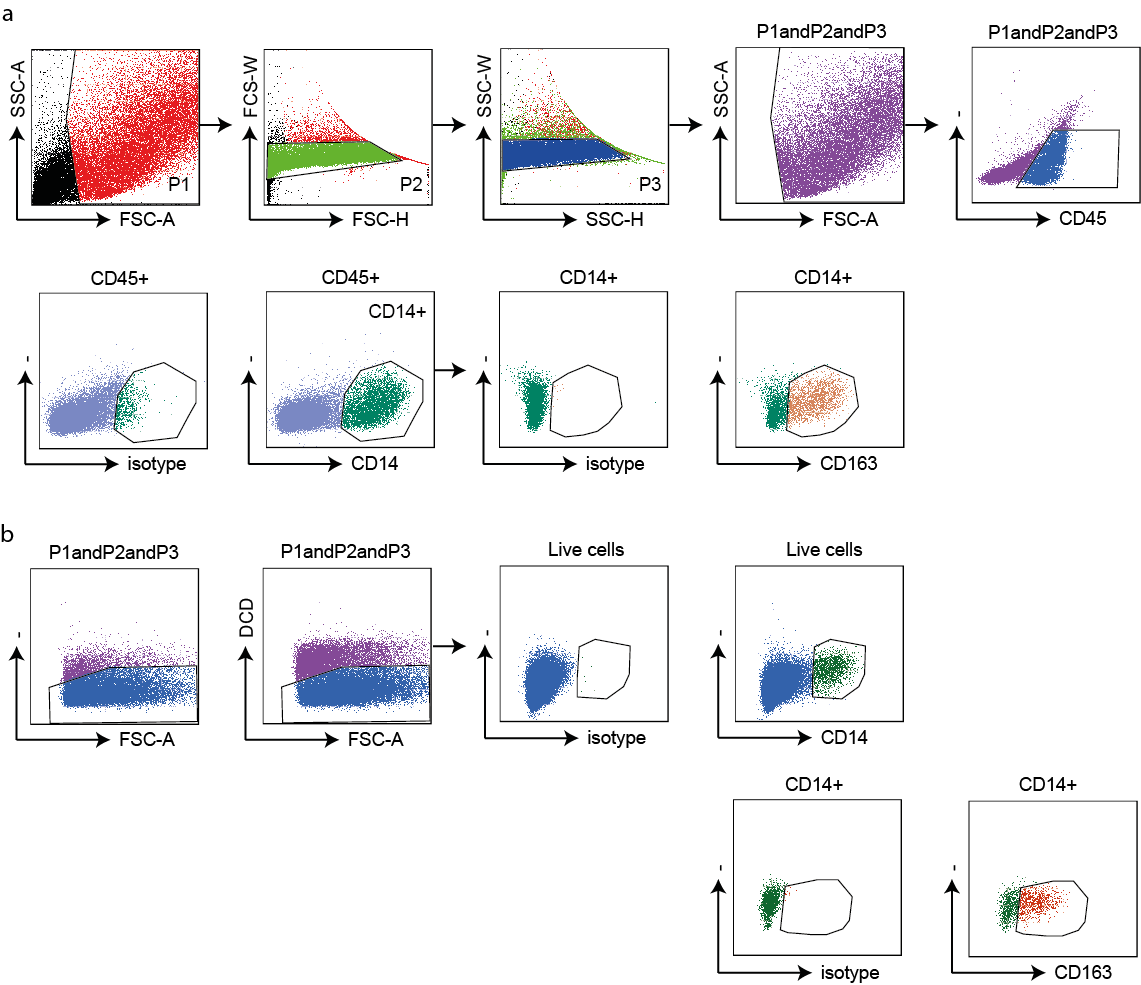


**Figure S3. Gating strategy**

Gating strategy used for phenotypical stainings (a), a live gate based on FSC-A/SSC-A was set on the stromal vascular cell fraction, followed by two gates to exclude double cells. The intersection of this gate was used for gating hematopoietic cells (CD45^+^), after which CD3^+^ T cells or CD14^+^ macrophages were gated. Within the CD14^+^ population phenotypic markers were further analyzed. Alternatively, CD14^+^CD163^+^ or CD14^+^CD163^-^ cells were selected and phenotypic markers were further analyzed. Within the CD3^+^ T cell population the expression of CD4 and CD8 were analyzed. Gating strategy used for intracellular stainings (b), a live gate based on FSC-A/SSC-A was set, followed by two gates to exclude double cells (as depicted in a). Within the intersection of these gates, cells positive for dead cell discriminator were excluded. CD14^+^ cells in this live gate were further analyzed for cytokine production or CD14^+^CD163^+^ or CD14^+^CD163^-^ were gated to analyze cytokine production.

**Table S1. Patient characteristics for all figures.**

| Patient characteristics | Fig 1 and Fig S2 (N=82) | Fig 2 (N=44) | Fig 3 (N=62) | Fig 4 (N=20) |
| --- | --- | --- | --- | --- |
| Age, year, mean (SD) | 65 (9.8) | 67 (9.3) | 67 (10.1) | 70 (7.1) |
| Female, N (%) | 66 | 64 | 63 | 70 |
| BMI, kg/m^2^, mean (SD) | 30.2 (6.4) | 29.7 (5.6) | 29.8 (5.3) | 29.8 (4.0) |

**Table S2. Levels of TNFα in FCM and ACM.** Differences between BMI ≤ 25 group and BMI > 30 group determined with Mann-Whitney test are indicated with p-value.

|  | **TNFα** | | | | | | |
| --- | --- | --- | --- | --- | --- | --- | --- |
|  | BMI ≤ 25 (N=8-11) | | BMI >25 and ≤ 30 (N=7-8) | | BMI > 30 (N=9-11) | | p-value |
|  | median | (min – max) | median | (min – max) | median | (min – max) |  |
| FCM | 0.0 | (0.0 – 15.8) | 6.3 | (0.0 – 2173. 9) | 8.6 | (0.0 – 165.1) | 0.04 |
| ACM | 5.0 | (1.4 – 40.1) | 16.8 | (3.6 – 35.9) | 5.8 | (1.3 – 168.3) | 0.51 |

**Table S3. Levels of cytokines/chemokines in FCM.** Differences between BMI ≤ 25 group and BMI > 30 group determined with Mann-Whitney test are indicated with p-value. The Bonferroni adjusted p-value indicating significance is < 0.001.

|  | **FCM** | | | | | | |
| --- | --- | --- | --- | --- | --- | --- | --- |
| Cytokines (pg/ml) | BMI ≤ 25 (N=8) | | BMI >25 and ≤ 30 (N=7) | | BMI > 30 (N=9) | | p-value |
|  | median | (min – max) | median | (min – max) | median | (min – max) |  |
| EGF | 0.0 | (0.0 – 5.7) | 3.4 | (0.0 – 10.3) | 5.2 | (0.0 – 10.3) | 0.09 |
| Eotaxin | 26.9 | (14.9 – 70.2) | 51.3 | (26.7 – 80.5) | 58.2 | (0.0 – 73.8) | 0.07 |
| FGF-2 | 184.9 | (64.7 – 1313.9) | 182.4 | (55.8 – 505.4) | 283.1 | (106.4–1150.2) | 0.54 |
| FLT3L | 12.5 | (0.0 – 18.7) | 15.9 | (4.8 – 40.8) | 17.4 | (0.0 – 28.7) | 0.19 |
| Fractalkine | 170.0 | (58.5 – 472.1) | 307.3 | (147.1 – 873.6) | 384.7 | (2.4 – 432.3) | 0.09 |
| G-CSF | 56.2 | (0.0 – 467.4) | 91.0 | (0.0 – 523.8) | 301.5 | (0.0 – 3890.7) | 0.15 |
| GM-CSF | 30.5 | (17.8 – 50.0) | 38.3 | (27.1 – 407.0) | 51.5 | (0.0 – 83.4) | 0.03 |
| GRO | 730.3 | (100.3 – 1157.9) | 1877.0 | (656.3 – 8925.5) | 2515.0 | (0.0 – 9158.9) | 0.23 |
| IFNα | 0.0 | (0.0 – 1.3) | 0.0 | (0.0 – 19.1) | 0.0 | (0.0 – 6.5) | 0.60 |
| IFNγ | 4.8 | (0.0 – 22.8) | 6.2 | (4.0 – 47.5) | 12.7 | (0.0 – 23.8) | 0.19 |
| IL1α | 0.0 | (0.0 – 5.7) | 9.9 | (0.0 – 171.8) | 2.8 | (0.0 – 18.3) | 0.26 |
| IL1β | 0.0 | (0.0 – 7.9) | 4.7 | (0.0 – 1318.5) | 6.2 | (0.0 – 33.1) | 0.07 |
| IL1RA | 19.3 | (0.0 – 98.7) | 35.0 | (11.4 – 1705.4) | 23.8 | (0.0 – 566.4) | 0.59 |
| IL2 | 0.0 | (0.0 – 0.0) | 0.0 | (0.0 – 5.2) | 0.0 | (0.0 – 0.0) | 0.99 |
| IL3 | 0.0 | (0.0 – 0.0) | 0.0 | (0.0 – 0.0) | 0.0 | (0.0 – 0.0) | 0.99 |
| IL4 | 0.0 | (0.0 – 0.0) | 0.0 | (0.0 – 8.9) | 0.0 | (0.0 – 11.1) | 0.21 |
| IL5 | 0.0 | (0.0 – 0.6) | 0.0 | (0.0 – 4.3) | 0.0 | (0.0 – 8.0) | 0.59 |
| IL6 | 754.8 | (189.3 – 2958.1) | 981.4 | (634.5 – 2935.8) | 1306.2 | (28.4 – 7628.7) | 0.09 |
| IL7 | 4.6 | (1.0 – 12.1) | 5.4 | (2.7 – 34.3) | 14.1 | (0.0 – 31.0) | 0.06 |
| IL8 | 1131.0 | (306.2 – 7708.9) | 2433.7 | (1126.1 – 9513.0) | 1945.7 | (20.5 – 4123.6) | 0.04 |
| IL9 | 0.0 | (0.0 – 0.0) | 0.0 | (0.0 – 1.2) | 0.0 | (0.0 – 0.6) | 0.99 |
| IL10 | 4.5 | (0.0 – 12.1) | 7.9 | (4.2 – 788.1) | 13.3 | (0.0 – 192.4) | 0.06 |
| IL12p40 | 4.1 | (0.0 – 7.9) | 5.4 | (2.8 – 25.8) | 2.8 | (0.0 – 25.8) | 0.83 |
| IL12p70 | 4.8 | (0.0 – 11.0) | 5.1 | (3.4 – 11.4) | 5.5 | (0.0 – 13.5) | 0.31 |
| IL13 | 0.0 | (0.0 – 0.0) | 0.0 | (0.0 – 6.4) | 0.0 | (0.0 – 3.1) | 0.99 |
| IL15 | 0.0 | (0.0 – 0.0) | 0.0 | (0.0 – 6.1) | 0.0 | (0.0 – 4.0) | 0.99 |
| IL17 | 0.0 | (0.0 – 0.0) | 0.0 | (0.0 – 4.0) | 0.0 | (0.0 – 0.0) | 0.99 |
| IP10 | 42.0 | (0.0 – 242.8) | 316.2 | (73.9 – 1483.6) | 63.6 | (0.0 – 709. 4) | 0.81 |
| MCP1 | 4468.0 | (0.0 – 10067.9) | 9023.6 | (0.0 – 10086.7) | 8929.9 | (86.1 – 9799.0) | 0.27 |
| MCP3 | 2.2 | (0.0 – 41.1) | 18.3 | (0.0 – 90.9) | 33.1 | (0.0 – 102.6) | 0.03 |
| MDC | 37.2 | (9.6 – 219.9) | 54.5 | (11.6 – 224.7) | 47.6 | (0.0 – 940.9) | 0.69 |
| MIP1α | 17.0 | (5.4 – 52.2) | 77.9 | (21.8 – 527.8) | 82.7 | (0.0 – 637.6) | 0.20 |
| MIP1β | 20.7 | (0.0 – 143.2) | 103.1 | (38.0 – 1031.0) | 68.7 | (0.0 – 248.9) | 0.23 |
| PDGF-AA | 10.9 | (3.4 – 16.5) | 16.4 | (8.7 – 35.0) | 18.0 | (1.0 – 25.0) | 0.24 |
| PDGF-ABBB | 0.0 | (0.0 – 0.0) | 0.0 | (0.0 – 0.0) | 0.0 | (0.0 – 15.5) | 0.99 |
| RANTES | 6.6 | (0.0 – 20.5) | 18.1 | (3.3 – 673.6) | 10.5 | (0.0 – 62.5) | 0.47 |
| sCD40L | 0.0 | (0.0 – 28.0) | 0.0 | (0.0 – 33.5) | 0.0 | (0.0 – 15.8) | 0.64 |
| sIL2-RA | 3.3 | (0.0 – 6.1) | 2.8 | (0.0 – 48.7) | 0.0 | (0.0 – 18.7) | 0.74 |
| TGFα | 0.0 | (0.0 – 0.5) | 0.0 | (0.0 – 3.3) | 0.0 | (0.0 – 0.0) | 0.47 |
| TNFβ | 0.0 | (0.0 – 3.5) | 0.0 | (0.0 – 3.5) | 0.0 | (0.0 – 3.5) | 0.58 |
| VEGF | 45.0 | (0.0 – 664.4) | 81.0 | (41.5 – 477.2) | 88.7 | (0.0 – 428.3) | 0.12 |

**Table S4. Levels of cytokines/chemokines in ACM.** Differences between BMI ≤ 25 group and BMI > 30 group determined with Mann-Whitney test are indicated with p-value. The Bonferroni adjusted p-value indicating significance is < 0.001.

|  | **ACM** | | | | | | |
| --- | --- | --- | --- | --- | --- | --- | --- |
| Cytokines (pg/ml) | BMI ≤ 25 (N=11) | | BMI >25 and ≤ 30 (N=8) | | BMI > 30 (N=11) | | p-value |
|  | median | (min – max) | median | (min – max) | median | (min – max) |  |
| EGF | 0.0 | (0.0 – 6.4) | 0.0 | (0.0 – 12.5) | 0.0 | (0.0 – 0.0) | 0.48 |
| Eotaxin | 9.7 | (0.0 – 30.2) | 20.8 | (9.2 – 34.1) | 8.9 | (0.0 – 41.0) | 0.99 |
| FGF-2 | 50.8 | (0.0 – 151.5) | 58.2 | (10.4 – 470.6) | 49.8 | (0.0 – 578.1) | 0.57 |
| FLT3L | 4.0 | (0.0 – 11.2) | 4.0 | (0.0 – 13.5) | 0.0 | (0.0 – 19.0) | 0.40 |
| Fractalkine | 17.0 | (0.0 – 174.3) | 64.5 | (32.6 – 370.8) | 32.6 | (0.0 – 229.9) | 0.48 |
| G-CSF | 67.7 | (3.9 – 556.1) | 144.6 | (17.5 – 1043.1) | 100.6 | (0.0 – 648.4) | 0.85 |
| GM-CSF | 3.4 | (0.5 – 40.1) | 13.0 | (0.0 – 59.9) | 4.8 | (0.7 – 36.0) | 0.61 |
| GRO | 254.8 | (118.6 – 2379.3) | 1306.4 | (117.2 – 2936.4) | 880.3 | (103.0 – 3663.5) | 0.40 |
| IFNα | 2.0 | (0.0 – 33.7) | 5.3 | (0.0 – 80.0) | 2.5 | (0.0 – 9.9) | 0.73 |
| IFNγ | 2.5 | (0.0 – 21.3) | 4.6 | (0.0 – 61.6) | 0.0 | (0.0 – 10.7) | 0.32 |
| IL1α | 0.0 | (0.0 – 3.2) | 0.0 | (0.0 – 4.6) | 0.0 | (0.0 – 6.7) | 0.48 |
| IL1β | 0.4 | (0.0 – 3.4) | 2.6 | (0.0 – 3.9) | 0.8 | (0.0 – 6.2) | 0.29 |
| IL1RA | 0.0 | (0.0 – 15.5) | 8.9 | (0.0 – 24.0) | 2.5 | (0.0 – 31.4) | 0.77 |
| IL2 | 0.0 | (0.0 – 1.1) | 0.3 | (0.0 – 1.4) | 0.0 | (0.0 – 2.0) | 0.68 |
| IL3 | 0.0 | (0.0 – 5.7) | 0.0 | (0.0 – 4.8) | 0.0 | (0.0 – 5.2) | 0.99 |
| IL4 | 0.0 | (0.0 – 0.0) | 0.0 | (0.0 – 0.0) | 0.0 | (0.0 – 14.5) | 0.04 |
| IL5 | 0.0 | (0.0 – 0.0) | 0.0 | (0.0 – 0.1) | 0.0 | (0.0 – 0.6) | 0.21 |
| IL6 | 142.7 | (28.9 – 979.2) | 760.2 | (44.4 – 1737.7) | 300.0 | (37.2 – 2854.8) | 0.52 |
| IL7 | 0.0 | (0.0 – 32.9) | 4.1 | (0.0 – 61.1) | 0.0 | (0.0 – 15.7) | 0.99 |
| IL8 | 670.5 | (98.9 – 5468.8) | 2022.5 | (145.5 – 5221.9) | 1543.8 | (328.5 – 9676.4) | 0.27 |
| IL9 | 0.0 | (0.0 – 0.0) | 0.0 | (0.0 – 0.0) | 0.0 | (0.0 – 0.0) | 0.99 |
| IL10 | 1.0 | (0.0 – 21.9) | 5.2 | (0.0 – 57.6) | 0.0 | (0.0 – 10.2) | 0.15 |
| IL12p40 | 0.0 | (0.0 – 12.5) | 0.9 | (0.0 – 26.1) | 0.0 | (0.0 – 2.9) | 0.59 |
| IL12p70 | 0.0 | (0.0 – 5.5) | 0.0 | (0.0 – 8.1) | 0.0 | (0.0 – 4.8) | 0.66 |
| IL13 | 0.0 | (0.0 – 1.2) | 0.0 | (0.0 – 1.3) | 0.0 | (0.0 – 1.8) | 0.25 |
| IL15 | 0.0 | (0.0 – 1.9) | 0.0 | (0.0 – 0.0) | 0.0 | (0.0 – 1.9) | 0.76 |
| IL17 | 0.0 | (0.0 – 0.0) | 0.0 | (0.0 – 0.0) | 0.0 | (0.0 – 2.1) | 0.99 |
| IP10 | 10.5 | (0.0 – 294.1) | 51.9 | (0.0 – 122.5) | 18.1 | (0.0 – 3768.0) | 0.50 |
| MCP1 | 391.5 | (138.7 – 7492.1) | 825.5 | (108.0 – 2380.8) | 582.9 | (51.2 – 9260.7) | 0.75 |
| MCP3 | 0.0 | (0.0 – 20.6) | 16.3 | (0.0 – 38.0) | 0.0 | (0.0 – 65.1) | 0.49 |
| MDC | 0.0 | (0.0 – 6.4) | 1.6 | (0.0 – 61.1) | 0.0 | (0.0 – 9.3) | 0.67 |
| MIP1α | 18.8 | (0.0 – 67.2) | 42.2 | (11.6 – 71.5) | 29.0 | (0.0 – 293.4) | 0.56 |
| MIP1β | 0.0 | (0.0 – 15.4) | 21.1 | (0.0 – 36.1) | 3.0 | (0.0 – 61.8) | 0.24 |
| PDGF-AA | 2.7 | (0.0 – 7.8) | 4.6 | (0.0 – 13.3) | 4.0 | (0.0 – 9.6) | 0.19 |
| PDGF-ABBB | 0.0 | (0.0 – 7.5) | 0.0 | (0.0 – 7.5) | 0.0 | (0.0 – 5.7) | 0.74 |
| RANTES | 0.5 | (0.0 – 6.1) | 3.9 | (0.0 – 6.7) | 1.5 | (0.0 – 53.9) | 0.63 |
| sCD40L | 0.0 | (0.0 – 7.0) | 0.0 | (0.0 – 7.0) | 0.0 | (0.0 – 21.5) | 0.99 |
| sIL2-RA | 0.0 | (0.0 – 4.8) | 0.0 | (0.0 – 13.8) | 0.0 | (0.0 – 4.6) | 0.74 |
| TGFα | 0.5 | (0.0 – 1.4) | 0.0 | (0.0 – 1.4) | 0.0 | (0.0 – 2.0) | 0.02 |
| TNFβ | 0.0 | (0.0 – 13.6) | 0.0 | (0.0 – 39.5) | 0.0 | (0.0 – 0.0) | 0.99 |
| VEGF | 17.5 | (0.0 – 50.8) | 53.7 | (17.2 – 105.0) | 14.9 | (0.0 – 139.3) | 0.59 |
